# Assessing the household financial burden associated with the chronic non-communicable diseases in a rural district of Vietnam

**DOI:** 10.3402/gha.v5i0.18892

**Published:** 2012-12-20

**Authors:** Hoang Van Minh, Bach Xuan Tran

**Affiliations:** Department of Health Economics, Center for Health System Research, Institute for Preventive Medicine and Public Health, Hanoi Medical University, Hanoi, Vietnam

**Keywords:** chronic non-communicable diseases, out-of-pocket expenditure, financial burden, rural, Vietnam

## Abstract

**Background:**

While there is accumulated evidence showing the rapid rise of the burden caused by non-communicable diseases (NCDs) in Vietnam, information on the extent to which households in the country suffer financial catastrophe or impoverishment caused by the diseases is still largely lacking. This paper aims to examine the self-reported prevalence of major chronic diseases among a population in rural Vietnam and to analyse the household financial burden associated with these diseases.

**Methods:**

A cross-sectional survey of 800 randomly selected households was carried out in Vo Nhai District, Thai Nguyen Province, in 2010. Face-to-face interviews were conducted with key informants of selected households on diagnosed chronic NCDs, health care utilization and health expenditure of all household members. The World Health Organization's definitions of catastrophic expenditure and impoverishment were used. Both descriptive and analytical statistics were applied.

**Results:**

The prevalence of chronic NCDs in households and individuals was 29.3 and 33.4%, respectively. The catastrophic health expenditure and impoverishment rates among the households who have at least one member with a chronic disease were 14.6 and 7.6%, respectively. These rates were significantly higher than the corresponding figures among the households whose members were free from the diseases (4.2 and 2.3%, respectively). The odds of experiencing catastrophic health expenditure and impoverishment among the household with NCD patients were 3.2 and 2.3 times greater than that of other households.

**Conclusion:**

Findings from this study indicate that the epidemiological and household financial burdens caused by chronic diseases in Vietnam are now substantial and need immediate mitigation measures.

The burden of chronic non-communicable diseases (NCDs) is increasing worldwide, especially in developing countries. Of the 57 million deaths that occurred globally in 2008, 36 million – accounting for almost two thirds – were due to chronic NCDs (mainly cardiovascular diseases, cancers, diabetes and chronic lung diseases) (1). Chronic NCDs not only cause premature death but also have major adverse effects on the quality of life of affected individuals and create large adverse economic effects on families, communities and societies (2, 3).

Vietnam is undergoing a rapid epidemiological transition that resulted in an increased number of chronic NCD cases. Chronic diseases have been shown to be major causes of morbidity and mortality in hospitals for the whole country. Hospital admissions due to chronic NCD diseases increased from 39% in 1986 to 66.2% in 2008 and chronic diseases deaths rose from 42% in 1986 to 63.3% in 2009 (4). Other community-based studies also proved that chronic NCDs have already been one of the leading causes of both morbidity (5) and mortality (6, 7) among Vietnamese populations.

The health system in Vietnam is a mixed public–private provider system, in which the public system still plays a key role in health care, especially in prevention, research and training. The public health care system in Vietnam is now organized into three levels (Ministry of Health, Provincial Health Services, District Health Facilities, and Commune Health Centres, including Village Health Workers). Vietnam is now using three main options to finance national health care expenditure, including the state budget, insurance contributions and direct out-of-pocket (OOP) payments by service users (8). Of the health financing sources, the state budget plays a critical role in protecting public health, accounting for approximately 25% of total health expenditure (9). National health insurance was introduced in Vietnam in 1992 and now has two national insurance schemes – a compulsory scheme and a voluntary scheme. The coverage of health insurance in Vietnam increased from 16% of the population in 2002 to 60% in 2010. However, health insurance contributions only accounted for 18% of total health expenditures in 2009 (10). The Government of Vietnam is committed to achieving full population health insurance coverage by 2020 (11). Another relatively large financial flow is household's direct OOP payments, partially as a result of the hospital fee policy introduced in 1989 following the Decision No. 45/HDBT dated 24 April 1989 by the Government of Vietnam, which allowed hospitals to recover costs through user fees. Direct OOP payments for health care refer to the expenditures directly made by households when they use services, primarily for the purchase of drugs, payment of hospital user fees, diagnostic service fees and other indirect expenses related to seeking medical care at state or private facilities (including self-medication) (8). In Vietnam, the OOP payments as a share of the total health expenditure have been always high, ranging from 50% to 70% (8).

**Policy recommendations**

The findings from this study indicate that the household financial burdens caused by chronic non-communicable diseases in Vietnam are substantial. The following points summarise the key policy recommendations:

- More attention should be paid on prevention and control of chronic non-communicable diseases in Vietnam.
- Interventions should be comprehensive and integrated, including both primary and secondary approaches, as well as policy-level involvements.
- As health insurance was shown to have some impact on financial protection, expanding coverage of health insurance in all three dimensions (breadth, height and depth) should be a priority policy action.
- Reforming provider payment methods could also be a good option to enhance the financial protection against chronic diseases in Vietnam.

While there is accumulated evidence showing the rapid rise of the burden caused by NCDs in Vietnam, information on the extent to which households in the country suffer financial catastrophe or impoverishment caused by the diseases is still largely lacking. This paper aims to examine the self-reported prevalence of major chronic diseases among a population in rural Vietnam and to analyse the household financial burden associated with the diseases.

## Methods

### Study design

This was a population-based cross-sectional study.

### Study setting

The study was carried out in Vo Nhai District, Thai Nguyen province. This is a mountainous district located 90 km north from Hanoi capital. The district has approximately 63,000 people living in 14 communes and one township. According to the local health statistics in 2010, the annual income per capita of the local population was approximately US$ 500. The proportion of poor households, as classified by local authorities (based on national poverty line using per capita income, area of land and assets household possessed), was approximately 10%. In terms of health status, chronic diseases were the leading causes of death in the district. Up to now, there has been no intervention to prevent and control chronic diseases in this setting.

### Sample size and sampling

A total of 800 households were randomly selected from the list of households in Vo Nhai provided by the district health centre. The same size was determined using the level of significance (α) of 0.05, relative precision (ɛ) of 0.3, the expected proportion of households incurring catastrophic health care payment (p) of 5.5% (finding from a study in Vietnam in 2011 (12)), and the anticipated non-response of 10%.

### Data collection

Face-to-face interviews were conducted with key informant of the selected households (usually head of the household). The interviews were carried out by trained surveyors from Hanoi Medical University using a structured questionnaire. The questionnaire was developed by the research team containing questions on diagnosed chronic NCDs (cardiovascular diseases, cancers, diabetes and chronic lung diseases) among household members, health care utilization and expenditure (during the last 12 months for inpatient care, during the last 4 weeks for outpatient cares and self-treatments). Information on the socio-economic condition of the household was also collected. Spot-checks and re-checks on sample data were conducted by the research team for quality control.

### Definitions

Respondents were asked if they had been told by a health worker that they had any of the following chronic conditions: cardiovascular, cancer, diabetes, chronic pulmonary diseases. The measures of catastrophic expenditure and impoverishment have been clearly described elsewhere (13, 14):

1. *OOP health payments* refer to the payments made by households at the point they received health services. OOP payments are the net of insurance reimbursement.
2. *Household's consumption expenditure* comprises both monetary and in-kind payment on all goods and services, and the money value of the consumption of homemade products.
3. *Household's capacity to pay* is defined as effective income remaining after basic subsistence needs have been met. Effective income is taken to be the total consumption expenditure of the household. Some households may report food expenditure that is lower than subsistence spending.
4. *Household subsistence spending* is the minimum requirement to maintain basic life in a society. A poverty line is used in the analysis as subsistence spending. Poverty line is defined as the food expenditure of the household whose food expenditure share of total household expenditure is at the 50th percentile in the country. In order to minimize measurement error, we use the average food expenditures of households whose food expenditure share of total household expenditure is within the 45th and 55th percentile of the total sample. Considering the economy scale of household consumption, the household equivalence scale is used rather than actual household size. The value of the parameter β has been estimated from previous studies based on 59 countries’ household survey data, and it equals 0.56.
5. *Catastrophic expenditure* occurs when a household's total OOP health payments equal or exceed 40% of household's capacity to pay.
6. *Impoverishment:* A non-poor household is impoverished by health payments when it becomes poor (when household expenditure is equal to or higher than subsistence spending but is lower than subsistence spending net of OOP health payments) after paying for health services

### Data analysis

Data were analysed using Stata statistical software version 10. Both descriptive and analytical statistics were applied. Logistic regressions were used to identify the socio-economic correlates of the catastrophic and poverty impacts of household's out-of-pocket health expenditure. The dependent variables included catastrophic health expenditure and impoverishment. The independent variables are socio-economic indicators such as sex of household head, household size, number of old people in the household, number of children under 6 years of age in the household, number of household members with insurance card and economic status classified by local Authorities (based on national poverty line using per capita income, area of land and assets household possessed). A significance level of *p*<0.05 was used.

### Ethical considerations

All human subjects in the study were asked for their informed consent before collecting data, and all had complete right to withdraw from the study at any time without having any threat.

## Results

Of the 800 selected households, 768 households, consisting of 3,378 people (46.8% male and 53.2% female), responded to the survey (response rate of 96%). On average, each studied household had 4.4 people. The proportion of poor households, as classified by local Authorities, was 9.9%. The annual per capita income of the study population was VND 9.7 million (around US$ 500).

Overall, 29.3% of the studied households had at least one member who had one or more chronic NCDs of interest. At individual level, 33.4% of respondents reported having at least one chronic NCDs (35.5% among men and 31.6% among women). The prevalence of self-reported chronic pulmonary disease, cardiovascular disease (including hypertension and stroke), diabetes and cancers among the studied participants were 24.9, 16.3, 0.2 and 0.3%, respectively (Table 1).

**Table 1:** Prevalence of self-reported chronic diseases among the study population

	Male (%)	Female (%)	Overall (%)
Any chronic disease	562 (35.5)	567 (31.6)	1129 (33.4)
Chronic pulmonary disease	349 (22.1)	496 (27.6)	841 (24.9)
Cardiovascular diseases	264 (16.7)	286 (15.9)	551 (16.3)
Diabetes	3 (0.2)	4 (0.2)	7 (0.2)
Cancer	2 (0.1)	9 (0.5)	10 (0.3)

As shown in Table 2, during the last 12 months, the rate of utilization of inpatient care services among households with and without NCD patients was 18.9 and 6.4%, respectively. The corresponding mean out-of-pocket payment for the inpatient care services was VND 5,760,000 and VND 2,472,000, respectively. During the last 4 weeks, the rate of utilization of outpatient care services among households with and without NCD patients was 52.1 and 32.3%, respectively. The corresponding mean out-of-pocket payment for the outpatient care services were VND 78,300 and VND 32,600, respectively. The differences in health service utilization rates and health payments between households with and without chronic NCD patients was statistically significant.

**Table 2:** Pattern of health care utilization and out-of-pocket payment for health care

	Households where there was at least one member with a chronic disease	Households without any person with chronic disease	*p*
Inpatient care services during the last 12 months			
Utilization: *n* (%)	43 (18.9)	35 (6.4)	0.00^a^
Mean out-of-pocket payment: VND	5,760,000	2,472,000	0.00^b^
Outpatient care services during the last 4 weeks			
Utilization: *n* (%)	117 (52.1)	175 (32.3)	0.00^a^
Mean out-of-pocket payment: VND	78,300	32,600	0.00^b^

a Note: Chi-squared test

b Mann–Whitney test.

Table 3 presents the patterns of catastrophic health expenditure and impoverishment among the studied households. The proportion of catastrophic health expenditure among the households where there was at least one member with a chronic disease and among those without any member with a disease were 14.6 and 4.2%, respectively. The impoverishment rates among the households where there was at least one member with a chronic disease and among those without any member with a disease were 7.6 and 2.3%, respectively. The differences in both the catastrophic health expenditure and the impoverishment rates between the households of chronic disease patients and other households were statically significant.

**Table 3:** Pattern of catastrophic health expenditure and impoverishment

	Households where there was at least one member with a chronic disease	Households without any person with chronic disease	*p* Chi-squared test
Catastrophic health expenditure: *n* (%)	33 (14.6)	23 (4.2)	0.00
Impoverishment: *n* (%)	17 (7.6)	12 (2.3)	0.00

Table 4 presents the results of logistic regression analysis of the correlates of catastrophic health expenditure. The significant correlates of the catastrophic health expenditure were: (1) Chronic disease: Households where there was at least one member with a chronic illness had a significantly higher rate of catastrophic expenditure (OR=3.2); (2) Household size: Having more people was significantly associated with a lower rate of catastrophic expenditure (OR=0.87); (3) Number of elderly people (aged 60 years and over) in the household: Having more elderly people was significantly associated with a higher proportion of catastrophic health expenditure (OR=1.2); (4) Economic status: Poor household were more likely to incur catastrophic expenditure for health (OR=2.6); and (5) Insurance status: Having at least one person with health insurance was associated with a slightly lower rate of catastrophic expenditure (OR=0.87).

**Table 4:** Correlates of catastrophic health expenditure

	OR	*p*
Having at least one member with a chronic illness		
Yes	3.2	0.00
No	1	
Sex of the household's head		
Male	0.87	0.66
Female	1	
Household size	0.79	0.00
Number of elderly people (aged 60 years and over) in the household		
Yes	1.32	0.00
No	1	
Number of children under 6 years in the household		
Yes	1.1	0.07
No	1	
Economic status		
Poor	2.6	0.02
Non-poor	1	
Having at least one person with health insurance card		
Yes	0.87	0.04
No	1	

Table 5 presents the results of logistic regression analysis of the correlates of impoverishment problem. The significant correlates of the impoverishment problem were: (1) Chronic disease: Households where there was at least one member with a chronic illness had a significantly higher rate of impoverishment (OR=2.3); 2) Household size: Having more people was significantly associated with a lower rate of impoverishment (OR=0.86); 3) Number of elderly people (aged 60 years and over) in the household: Having more elderly people was significantly associated with a higher proportion of impoverishment (OR=1.1); 4) Insurance status: Having at least one person with health insurance was associated with a slightly lower rate of impoverishment (OR=0.72).

**Table 5:** Correlates of impoverishment

	OR	p
Having at least one member with a chronic illness		
Yes	2.3	0.00
No	1	
Sex of the HH's head		
Male	0.79	0.79
Female	1	
Household size	0.86	0.00
Number of elderly people (aged 60 years and over) in the household		
Yes	1.1	0.00
No	1	
Number of children under 6 years in the household		
Yes	1.3	0.12
No	1	
Having at least one person with health insurance card		
Yes	0.72	0.04
No	1	

## Discussion

A high prevalence of self-reported chronic NCDs in studied households and individuals (29.3 and 33.4%, respectively) observed in this study proves the fact that chronic NCDs have already affected a large population in this setting. A study in another rural area in the North of Vietnam in 2007 reported a higher individual prevalence of chronic disease (39%), because that also included chronic joint problems (5). Several other studies also showed that the burden of disease from chronic diseases in Vietnam was substantial (15–18).

Of particular interest in this article is the household financial burden associated with chronic diseases. We have shown that when a household member has a chronic illness, he or she was forced to use more health services and, as a consequence, the household had to spend more of its OOP money on health care for that ill member. Many households in the study setting incurred a catastrophic level of health expenditure and/or were pushed into poverty because of health care payments. The catastrophic health expenditure and the impoverishment rates were higher among the households where there was at least one member with a chronic disease (14.6 and 7.6%, respectively) compared to the corresponding figures among the households who were free from the diseases (4.2 and 2.3%, respectively). The odds of catastrophic health expenditure and impoverishment among the household with NCD patients were 3.2 and 2.3 times greater than that for other households.

There have been very few studies on this area from Vietnam. A study by Hien et al. in northern Vietnam in 2004 found that 19% of rural dwellers with diabetes had to sell assets, using savings or borrowing from neighbours to pay for health care costs (19). A study by Thuan et al. in a rural district in Vietnam in 2006 revealed that household expenditure on the treatment of chronic disease illnesses were also considerable (20). Wagstaff et al. found that Vietnamese households have not been able to hold their food and non-food consumption constant in the face of income reductions and extra medical care spending because of chronic illness (21). A recent study by WHO, using the National Living Standard Survey data, reported that the catastrophic health expenditure and the impoverishment rates due to general health care expenditure of the whole country in 2008 were 5.5 and 3.9%, respectively (12).

The household financial burden from chronic diseases was also pronounced by international publications. A recent review by Saksena et al. documented findings from studies on the impact of OOP payments for treatment of NCDs from various developing countries such as Pakistan in 2004 (22), Burkinafaso in 2006 (23), Kenya in 2007 (24), China in 2009 (25), China in 2010 (26, 27), India in 2008 (28) and India in 2012 (29). The authors concluded that the households with chronic disease patients had to spend a substantial share of their income on care for these diseases, and many households experienced catastrophic health expenditure and impoverishment as a result of their health spending (30). A review by Engelgau et al. also confirmed that the financial risks from chronic diseases put on households in developing countries were significant (31).

In this study, we found that the financial burden on poor households was generally higher than the burden on richer households. This is similar to findings from previous studies in India in 2008 (28) and in China in 2009 (25). Engelgau et al. also demonstrated that the household financial burden from chronic diseases impacted more on the poor and vulnerable populations (31). We also found that households with more elderly people had a higher probability of encountering catastrophic payment. While Vietnam is undergoing demographic transition and experiencing rapid population ageing, there is still no specific policy on health for the elderly (32, 33). More attention on health care for elderly people in Vietnam is needed in the future.

Although financial protection is the most important aspect of health insurance coverage, we found little impact of health insurance on protecting people from catastrophic payment and impoverishment. Most of the studies on impacts of health insurance in Vietnam consistently found that insurance have only a modest effect on reducing OOP payments (34–38). The modest impact of insurance on financial protection reflects the fact that insured patients did co-pay for a high share of costs for medical care, especially for chronic disease care services.

We need to note some limitations of this study. First, the cross-sectional nature of the data only allowed us to examine the short-term impacts of household direct OOP payments. Secondly, we were just able to observe the OOP payments for health, catastrophic health expenditure and impoverishment proportions among the households where there was at least one member with a chronic disease, and also figures among the households whose members were free from the diseases. We did not know whether or not the payments of the households with chronic disease patients were actually for the chronic disease care or for other health services.
